# The FDA initiatives for personalized medicine

**DOI:** 10.1186/1878-5085-5-S1-A6

**Published:** 2014-02-11

**Authors:** Olive Joy Wolfe

**Affiliations:** 1 Clinical consultants, Inc., Glen Rock, NJ07410, USA

## 

The FDA continues to be the Leader on the scientific front to build the regulatory infrastructure necessary to support the development of personalized targeted therapies. To this end the FDA has drafted guidance documents for industry to follow for both diagnostic and therapeutic research trials. For example, there has been an increase in companion diagnostics, the tests being used to determine whether or not a particular therapy may work for a patient. To address these issues, the FDA has published a draft guidance: *In Vitro Companion Diagnostic Devices* in July 2011, to communicate to Industry how the FDA defines these devices and what the regulatory requirements are for them. These guidelines should help to facilitate the process but will also help determine that the right patient received the right treatment for them. In addition, the FDA is reaching out to drug developers, academic investigators, patient groups, statistical and methodological experts and ethicists through a series of meetings to achieve a mutual understanding of the necessary steps to be taken when an investigational drug being studied for a serious disease with no alternative treatment option shows “exceptional” promise. Through the coordination of both arms of the FDA’s regulatory centers, CDRH (diagnostic approvals) and CBER and CDER (drugs, biologics and cell-based therapies), a more expedited pathway for approval will be possible. Draft guidances on any of these issues from the outcome of the above meetings as well as the FDA’s strategic plans to enhance their statistical and computational models will be issued by CDER. The most recent developments on any of the above will be addressed.

